# Supplementary material for: Acetylation reprograms MITF target selectivity and residence time
Source: Nat Commun. 2023 Sep 28;14:6051. doi: 10.1038/s41467-023-41793-7 (PMC10539308; doi:10.1038/s41467-023-41793-7)
Supplement: Supplementary file 1 — Supplementary Information [file 41467_2023_41793_MOESM1_ESM.pdf]

Query	Observed	Mr(expt)	Mr(calc)	ppm	M	Score	Expect	Rank	U	Peptide	
929	531.3503	530.3431	530.3428	0.52	0	15	0.52	1	U	K.GTILK.A	
1124	323.6616	645.3086	645.3082	0.60	0	15	0.4	2	U	K.DLENR.Q	
1141	651.4297	650.4224	650.4228	-0.54	0	14	0.75	1	U	R.HLLLR.V	
1142	**326.2187**	**650.4228**	**650.4228**	**0.10**	**0**	**30**	**0.023**	1	**U**	**R.HLLLR.V**	
1147	657.3239	656.3166	656.3170	-0.58	0	27	0.2	1	U	K.EAFYK.F	
1148	329.1658	656.3171	656.3170	0.24	0	23	0.13	1	U	K.EAFYK.F	
1227	778.3846	777.3773	777.3769	0.47	0	23	0.43	1	U	R.FNINDR.I	
1264	**412.2186**	**822.4227**	**822.4236**	**-1.03**	**0**	**53**	**0.0017**	1	**U**	**K.ASVDYIR.K**	
1277	**417.6743**	**833.3340**	**833.3338**	**0.24**	**0**	**34**	**0.0071**	1	**U**	**K.SNDPDMR.W**	
1278	834.3420	833.3347	833.3338	1.14	0	15	0.62	1	U	K.SNDPDMR.W	
1326	870.5289	869.5217	869.5222	-0.63	0	14	0.47	1	U	K.ELGTLIPK.S	
1365	467.7505	933.4864	933.4780	8.91	1	32	0.24	1	U	R.RFNINDR.I	
1390	1001.5779	1000.5706	1000.5705	0.064	1	20	0.072	1	U	R.WNKGTILK.A + Acetyl (K)	K243
1391	501.2928	1000.5711	1000.5705	0.54	1	31	0.059	1	U	R.WNKGTILK.A + Acetyl (K)	K243
1397	505.7544	1009.4942	1009.4941	0.095	0	18	0.37	1	U	K.DNHNLIER.R	
1399	337.5054	1009.4943	1009.4941	0.20	0	10	2.8	9	U	K.DNHNLIER.R	
1445	**552.2819**	**1102.5493**	**1102.5441**	**4.74**	**0**	**59**	**0.00037**	1	**U**	**R.VQELEMQAR.A**	
1451	**556.3572**	**1110.6998**	**1110.7012**	**-1.27**	**1**	**45**	**0.00088**	1	**U**	**R.IKELGTLIPK.S**	
1452	**560.2764**	**1118.5383**	**1118.5390**	**-0.61**	**0**	**58**	**0.001**	1	**U**	**R.VQELEMQAR.A + Oxidation (M)**	
1494	394.2067	1179.5983	1179.5996	-1.05	1	41	0.056	1	U	K.KDNHNLIER.R + Acetyl (K)	K206
1576	492.5385	1474.5935	1474.5929	0.43	0	12	1.2	2	U	R.AESECPGMNTHSR.A	
1577	**738.3408**	**1474.6671**	**1474.6728**	**-3.89**	**1**	**50**	**0.00011**	1	**U**	**K.EAFYKFEEQSR.A + Acetyl (K)**	K91
1619	**812.3873**	**1622.7601**	**1622.7610**	**-0.55**	**0**	**59**	**7.3e-05**	1	**U**	**R.ELTACIFPTESEAR.A**	
1620	541.9279	1622.7619	1622.7610	0.57	0	14	0.51	1	U	R.ELTACIFPTESEAR.A	
1625	**558.3051**	**1671.8933**	**1671.8944**	**-0.63**	**1**	**46**	**0.0021**	1	**U**	**R.HQVKQYLSTTLANK.H + Acetyl (K)**	K33
1690	**1054.0521**	**2106.0897**	**2106.0892**	**0.25**	**0**	**100**	**3.3e-08**	1	**U**	**R.AHGLSLIPSTGLCSPDLVNR.I**	

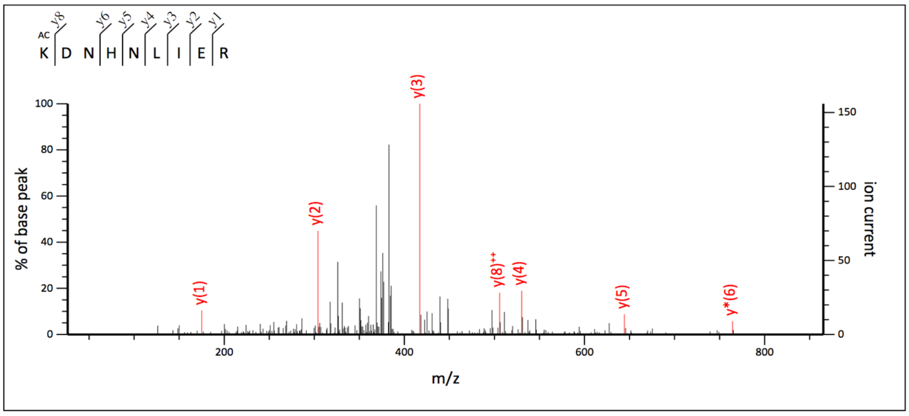

#	b	b++	b*	b*++	b0	b0++	Seq.	y	y++	y*	y*++	y0	y0++	#
1	171.1128	86.0600	154.0863	77.5468			K							9
2	286.1397	143.5735	269.1132	135.0602	268.1292	134.5682	D	1010.5014	505.7543	993.4748	497.2411	992.4908	496.7490	8
3	400.1827	200.5950	383.1561	192.0817	382.1721	191.5897	N	895.4744	448.2409	878.4479	439.7276	877.4639	439.2356	7
4	537.2416	269.1244	520.2150	260.6112	519.2310	260.1191	H	781.4315	391.2194	764.4050	382.7061	763.4209	382.2141	6
5	651.2845	326.1459	634.2580	317.6326	633.2739	317.1406	N	*644.3726*	322.6899	627.3461	314.1767	626.3620	313.6847	5
6	764.3686	382.6879	747.3420	374.1747	746.3580	373.6826	L	*530.3297*	265.6685	513.3031	257.1552	512.3191	256.6632	4
7	877.4526	439.2300	860.4261	430.7167	859.4421	430.2247	I	*417.2456*	209.1264	400.2191	200.6132	399.2350	200.1212	3
8	1006.4952	503.7513	989.4687	495.2380	988.4847	494.7460	E	*304.1615*	152.5844	287.1350	144.0711	286.1510	143.5791	2
9							R	*175.1190*	88.0631	158.0924	79.5498			1

Supplementary Fig. S1. MITF is acetylated at K206. Mass Spec. analysis of immunoprecipitated MITF ectopically expressed with p300 in HEK293 cells highlighting acetylated lysines (top) and MS/MS spectra (middle) showing b- and y-ion coverage of the Acetyl K206 peptides (bottom).

Louphrasitthiphol et al Figure S1

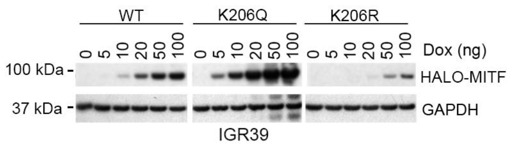

Supplementary Fig. S2, Inducible HALO-MITF IGR39 cells. Western blot of IGR39 cells in which expression of the HALO-MITF WT and K206 mutants are induced for 16 h using indicated amounts of doxycycline. To achieve similar levels of HALO-MITF WT and mutant expression in the SMT experiments shown in Supplementary Fig. S3., the amounts of doxycycline used were WT, 20 ng/ml; K206Q, 5 ng/ml; K206R, 100 ng/ml and the level of HALO-MITF induced corresponds to the representative blot shown here. The inducibility of HALO-MITF by doxycycline in IGR39 cells has been reproduced independently at least 5 times. Source data for western blots are shown in Supplementary Figure S6.

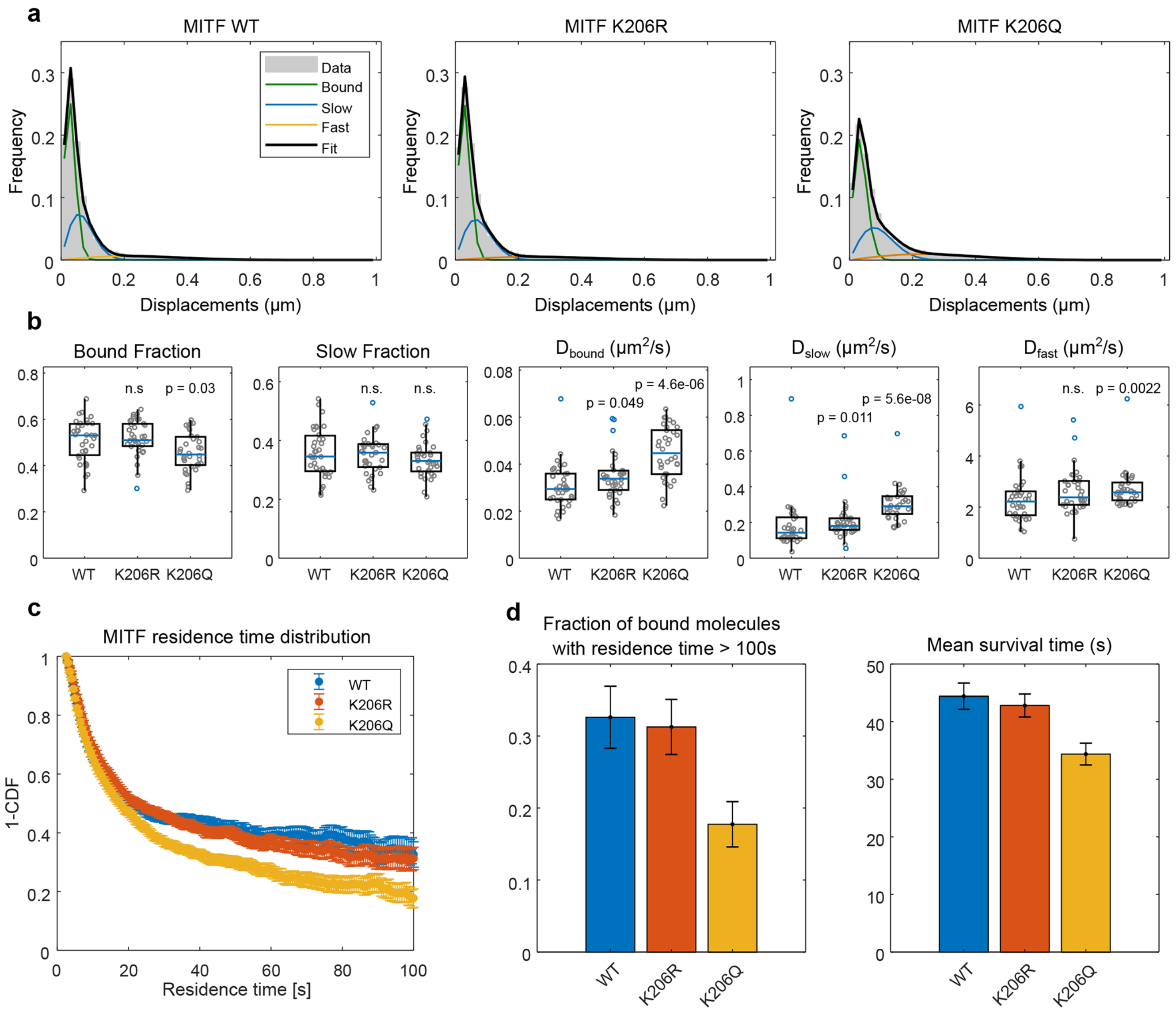

Supplementary Fig. S3, Live-cell single-molecule tracking of HALO-tagged MITF in IGR39 cells. **a.** Single molecule tracking movies collected at 100 fps tracks were extracted using the FiJi/ImageJ plugin TrackMate and analysed in terms of the distribution of single-molecule displacements between consecutive frames that was then fit with a three-component model (one immobile component and two diffusing components), to generate quantitative estimates for WT MITF and K206 mutants. **b.** Quantitative estimates derived from SMT using WT and K206 HALO-tagged MITF for the fraction of molecules in the bound and slow state, and of the diffusion coefficients of molecules belonging to each state. Each point represents a single cell, the blue line the median, and the box limits represent upper and lower quartiles, and whiskers extend between Q1 - 1.5 IQR and Q3 + 1.5 IQR, where IQR is the interquartile range. For Halo WT MITF, K206R and K206Q mutants respectively $N_{replicates} = 2$; N_{cells} = 35, 35, 34; N_{jumps}: 154969; 241707; 201391. Statistical test non-parametric Kruskal Wallis (two-sided). **c.** Slower movies (frame rate 2fps, laser exposure 200 ms) were acquired to calculate the distribution of residence times for immobile TF molecules, following photobleaching correction using data collected on H2B-HALO Tag (see methods). **d.** The fraction of detected bound molecules displaying a residence time longer than 100 s (left) and the (restricted) mean survival time (right). For panels **c,d**, $N_{replicates} = 2$; N_{cells}= 35, 35, 35; $N_{molecules}$: *3686; 3822, 4290* for WT MITF, K206R, K206Q, respectively; error bars represent SEM calculated by the jacknife approach described in the Methods section.

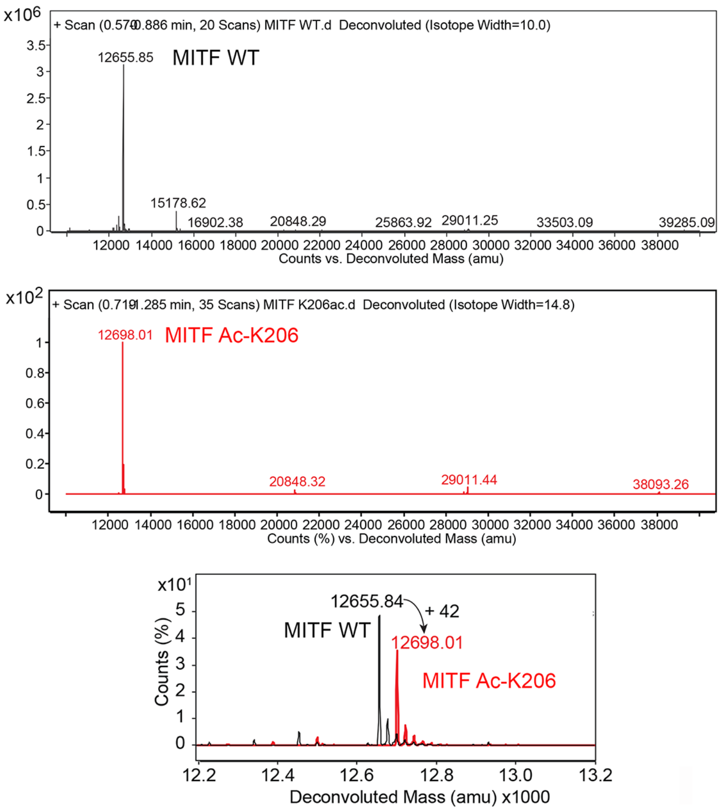

Supplementary Fig. S4, Mass Spec. spectra of bacterially expressed and purified MITF DNA binding domain. The spectra show non acetyl MITF (upper panel) and acetyl-K206 (middle panel) and the mass shift between the two (lower panel).

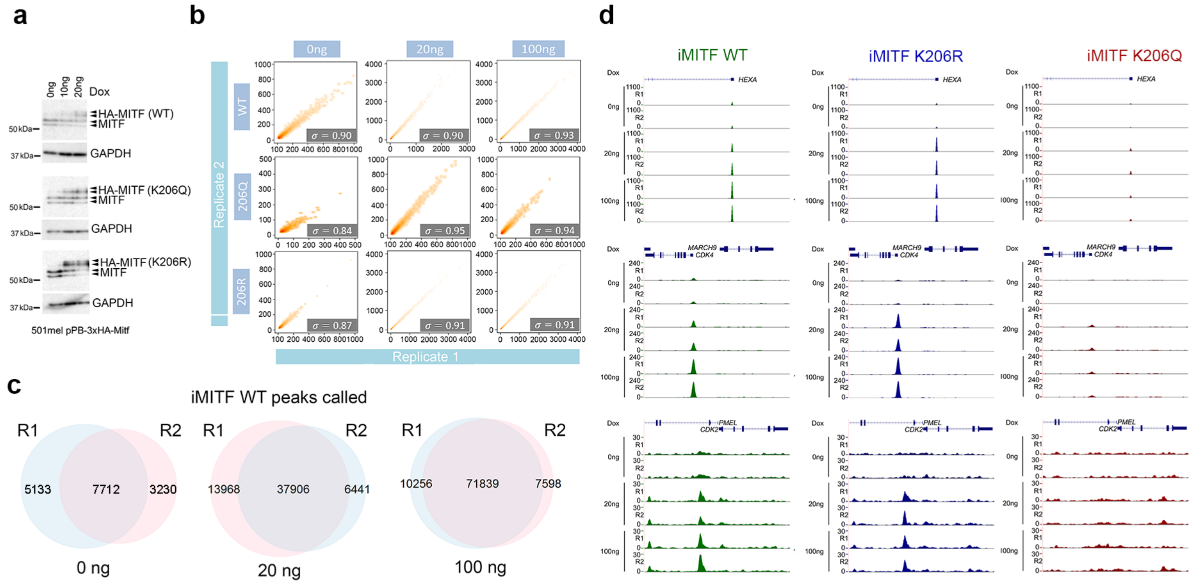

Supplementary Fig. S5, Genome-wide binding of MITF WT and K206 mutants. **a.** Western blot of 501mel cells stably expressing ectopic doxycycline inducible HA-WT and mutant MITF at 0, 10 and 20 ng doxycycline probed with anti-MITF antibody. MITF inducibility by Dox is highly reproducible and has been repeated at least 5 times. Source data are provided in Supplementary Fig. S6. **b.** Comparison of MITF WT and mutant ChIP-seq peak scores for each replicate at 0, 20 and 100 ng doxycycline. **c.** Numbers of peaks called for each replicate ChIP-seq for MITF WT at different doxycycline concentrations. **d.** UCSC genome-browser screenshots of ChIP-seq profiles of HA-MITF WT and mutants at different concentrations of doxycycline as indicated.

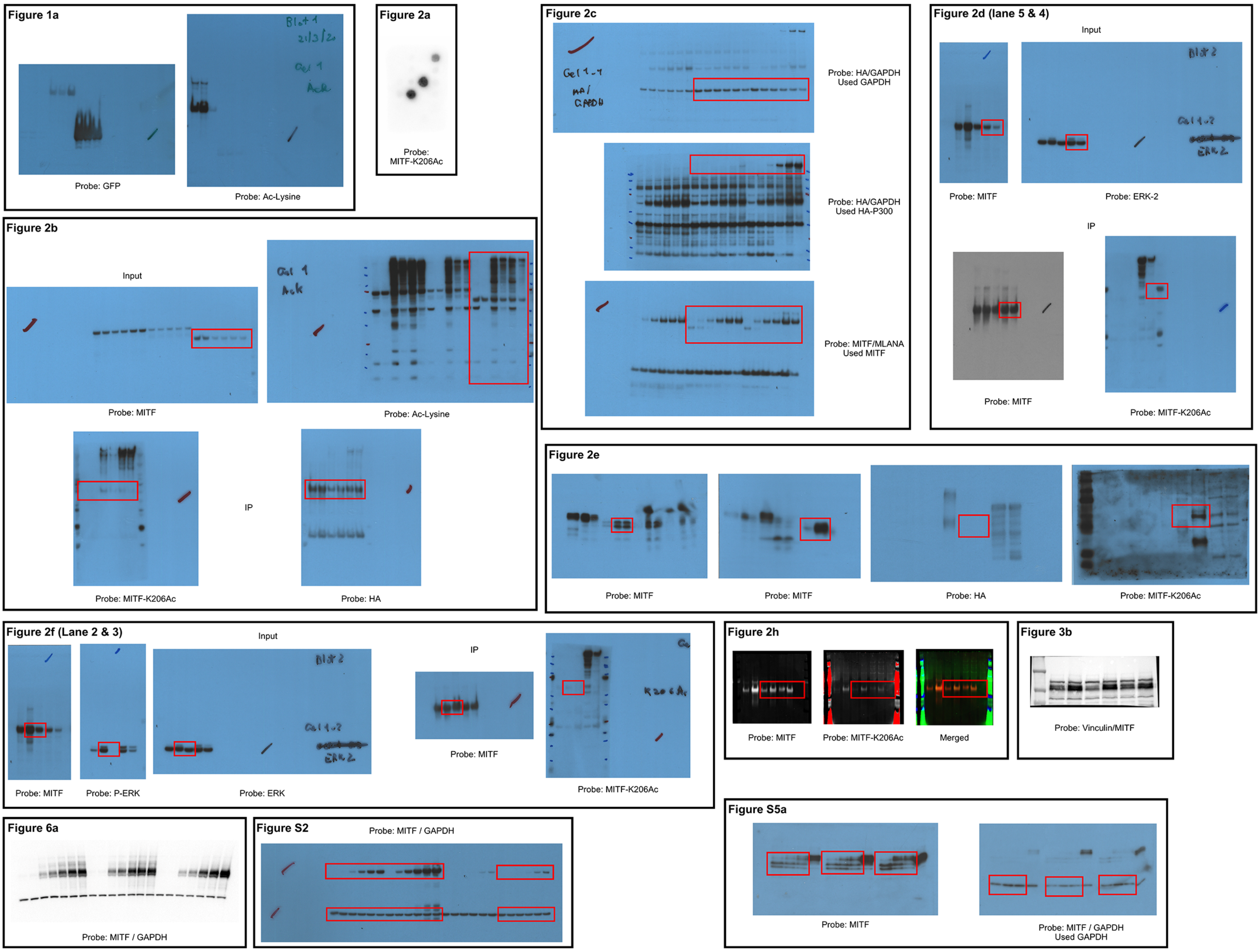

Supplementary Fig. S6, Source data for Western blots.
