# Supplementary material for: Acetylation reprograms MITF target selectivity and residence time
Source: Nat Commun. 2023 Sep 28;14:6051. doi: 10.1038/s41467-023-41793-7 (PMC10539308; doi:10.1038/s41467-023-41793-7)
Supplement: Supplementary file 3 — Reporting Summary [file 41467_2023_41793_MOESM3_ESM.pdf]

nature research

Corresponding author(s): Colin R Goding

Last updated by author(s): 09/08/2023

Reporting Summary

Nature Research wishes to improve the reproducibility of the work that we publish. This form provides structure for consistency and transparency in reporting. For further information on Nature Research policies, see Authors & Referees and the Editorial Policy Checklist .

Statistics

For all statistical analyses, confirm that the following items are present in the figure legend, table legend, main text, or Methods section.

n/a	Confirmed	
☐	☒	The exact sample size (*n*) for each experimental group/condition, given as a discrete number and unit of measurement
☐	☒	A statement on whether measurements were taken from distinct samples or whether the same sample was measured repeatedly
☐	☒	The statistical test(s) used AND whether they are one- or two-sided<br>*Only common tests should be described solely by name; describe more complex techniques in the Methods section.*
☐	☒	A description of all covariates tested
☐	☒	A description of any assumptions or corrections, such as tests of normality and adjustment for multiple comparisons
☐	☒	A full description of the statistical parameters including central tendency (e.g. means) or other basic estimates (e.g. regression coefficient) AND variation (e.g. standard deviation) or associated estimates of uncertainty (e.g. confidence intervals)
☐	☒	For null hypothesis testing, the test statistic (e.g. *F*, *t*, *r*) with confidence intervals, effect sizes, degrees of freedom and *P* value noted<br>*Give P values as exact values whenever suitable.*
☒	☐	For Bayesian analysis, information on the choice of priors and Markov chain Monte Carlo settings
☒	☐	For hierarchical and complex designs, identification of the appropriate level for tests and full reporting of outcomes
☒	☐	Estimates of effect sizes (e.g. Cohen's *d*, Pearson's *r*), indicating how they were calculated

Our web collection on statistics for biologists contains articles on many of the points above.

Software and code

Policy information about availability of computer code

Data collection	Image Lab 5.2.1 build 11 (Bio-Rad Laboratories)
Data analysis	STAR v 2.5.1b, Genome GRCh38 v23 (Dobin et al., 2013)<br><br>Homer v4.9.1, Genome hg38 v5.10 (Heinz et al., 2010)<br><br>ggplot2-v3.0.0 – under R-v3.5.1 https://CRAN.R-project.org/package=ggplot2<br><br>TreeView v1.1.6r4 (Saldanha, 2004)<br><br>PicardTools version 1.96, http://picard.sourceforge.net<br><br>MEME v5.0.1 (Machanick and Bailey, 2011)

For manuscripts utilizing custom algorithms or software that are central to the research but not yet described in published literature, software must be made available to editors/reviewers. We strongly encourage code deposition in a community repository (e.g. GitHub). See the Nature Research guidelines for submitting code & software for further information.

Data

Policy information about availability of data

All manuscripts must include a data availability statement. This statement should provide the following information, where applicable:

- Accession codes, unique identifiers, or web links for publicly available datasets
- A list of figures that have associated raw data
- A description of any restrictions on data availability

All the ChIP-seq raw, processed and bioinformatics pipelines involved are available at GSE137776.
https://www.ncbi.nlm.nih.gov/geo/query/acc.cgi?acc=GSE137776
These data were used to generate Fig 6b, 6c, 6d, 6e, 6f, 6g and Supplementary Fig. S5b, S5c, S5d

Raw unprocessed Western blots and their corresponding figures are included as Source Data in Supplemental Figure S6.
These blots have been used to generate Figure 1a, 2a, 2b, 2c, 2d, 2e, 2f, 2g, 2h, 3b, 6a, Supplementary Figure S2, S5a

Raw measurement for fluorescence anisotropy experiments used to generate Figure 4 are included in the Source Data Excel file.

Raw quantification of melanocytes in the zebrafish complementation experiments used to generate Figure 5 are included in the Source Data Excel file.

Field-specific reporting

Please select the one below that is the best fit for your research. If you are not sure, read the appropriate sections before making your selection.

☒ Life sciences ☐ Behavioural & social sciences ☐ Ecological, evolutionary & environmental sciences

For a reference copy of the document with all sections, see nature.com/documents/nr-reporting-summary-flat.pdf

Life sciences study design

All studies must disclose on these points even when the disclosure is negative.

Sample size

Single-molecule tracking (SMT) in 501mel cell line (Figure 2c, 2d, 2e, 2f)
SMT 100 fps - Nreplicates = 2; Ncells = 19, 30; 30; 30 Njumps: 130387; 245200; 210929; 172286, for Halo, WT MITF, K206R, K206Q respecitvely
SMT 2fps - Nreplicates = 2; Ncells= 30, 30, 30, 30, 35, 36; ; Nmolecules: 3836; 3429; 3219 ; 1185; 3809; 1225 for WT MITF, K206R, K206Q, Dbasic, USF1 and p53 respectively

Single-molecule tracking in IGR39 cell line (Supplementary Figure S3a, S3b, S3c, S3d)
SMT 100fps - Nreplicates = 2; Ncells = 35, 35, 34; Njumps: 154969; 241707; 201391 for WT MITF, K206R, K206Q respectively
SMT 2fps - Nreplicates = 2; Ncells= 35, 35, 35; Nmolecules: 3686; 3822, 4290; for WT MITF, K206R, K206Q respectively

Zebrafish complementation experiment - 7 experiments, 411 fish analysed, 20 for empty vector control, 132 for WT-Mitf, 201 for K201R and 127 for K201Q

Data exclusions

No individual data points for any given set of experiments have been exclude. All presented figures include all the data points for the given experiments.

Replication

Single-molecule tracking - Two independent replicates after final adjustment of doxycycline concentration to ensure similar levels of MITF for both 501mel and IGR39. Twice at the initial 20 ng doxycycline concentration (yielding the same conclusion between MITF WT, MITF K206Q and MITF K206R).

Fluorescence anisotropy - Three replicates per purified MITF protein

Zebrafish complementation experiment - Three replicates for each of the: No vector control, mitfa WT, mitfa K201R and mitfa K201Q

ChIP-seq experiments were performed as biological replicates. With the exception of MITF-WT which had been performed in at least 5 separate studies, each study include two biological replicates. Reported here are two biological replicate from one study.

Western blots (information also provided in figure legends):
Doxycycline titration of the MITF inducible cell lines corresponding to Figures 2c, 3b, 6a, S2, S5a: Data has been reproduced twice at the time of cell line establishment and on subsequent use for each and every cell line; 2-3 times at the fine tuning steps for Single-molecule tracking experiments shown in Fig. 3 and Fig. S3.

Fig. 1a has been performed twice using CBP/p300 with similar results, and once using GCN2

	Fig. 2 contains comprises multiple western blots performed under different conditions all aimed at confirming K206 acetylation detected by Mass Spectrometry.<br>Fig.2 b, d,e, have each been performed once, but in an experiment related to panel 2b, increased MITF acetylation has been observed using a p300 activator that was inhibited by A485; Fig. 2f has been repeated twice with similar results ;Fig. 2g has been performed once as presented, but repeated using S73A and S73A, S69A mutants with similar results. Fig. 2h has been repeated 3 times with similar results. Two replicates are shown.
Randomization	Randomization is not relevant to this study as the study investigate only one variable, acetylation status of MITF on K206 and the relevant molecular (Single-molecule tracking, fluorescence anisotropy and genome-wide binding) and biological consequence (zebrafish complementation) on MITF ability to function.
Blinding	Blinding was not relevant to this study as this study mechanistically investigate molecular and biological consequence of MITF K206 acetylation. The knowledge of MITF acetylation status (or the acetylation mimetics or non acetylable mimetics) cannot bias: group allocation (all mutants/K206-acetylated were subjected to identical investigation using fluorescence anisotropy, single molecule tracking and ChIP-seq), participants expectation (Fish embryo did not get to choose which plasmids they received) or observer bias (cell line for the SMT experiment, movie acquisitions and data analyses were conducts by three independent investigators located in two different countries).

Reporting for specific materials, systems and methods

We require information from authors about some types of materials, experimental systems and methods used in many studies. Here, indicate whether each material, system or method listed is relevant to your study. If you are not sure if a list item applies to your research, read the appropriate section before selecting a response.

Materials & experimental systems

n/a	Involved in the study	
☐	☒	Antibodies
☐	☒	Eukaryotic cell lines
☒	☐	Palaeontology
☐	☒	Animals and other organisms
☒	☐	Human research participants
☒	☐	Clinical data

Methods

n/a	Involved in the study	
☐	☒	ChIP-seq
☒	☐	Flow cytometry
☒	☐	MRI-based neuroimaging

Antibodies

Antibodies used	anti-HA (clone 12CA5) Supplier: Roche Catalog Number: 11666606001; RRID: AB_514506 Application: ChIP-seq Dilution: 120µg per ChIP-seq sample Application: Western blot Dilution: 1/10,000<br>anti-HA (clone HA-7) Supplier: Sigma-Aldrich Catalog Number: H3663; RRID: AB_262051 Application: Western blot Dilution: 1/5000<br>anti-HA (Clone 6E2) Supplier: Cell Signaling Technology Catalog Number: 2367; RRID: AB_2314619 Application: Western blot Dilution: 1/5000<br>anti-Acetylated-Lysine Supplier: Cell Signaling Technology Catalog Number: 9441; RRID: AB_331805 Application: Western blot Dilution: 1/1000<br>anti-MITF (Clone C5) Supplier: Millipore Catalog Number: MAB3747; RRID: AB_570596 Application: Western blot Dilution: 1/2000<br>anti-ERK 2 (Clone K-23) Supplier: Santa Cruz Biotechnology Catalog Number: sc-153; RRID: AB_2141293 Application: Western blot Dilution: 1/10,000<br>anti-ERK 2 (Clone D-2) Supplier: Santa Cruz Biotechnology Catalog Number: sc-1647; RRID: AB_627547 Application: Western blot Dilution: 1/3000<br>anti-Thr202/Tyr204, ppERK (Clone 197G2) Supplier: Cell Signaling Technology Catalog Number: 4377; RRID: AB_331775 Application: Western blot Dilution: 1/1000<br>anti-GAPDH (Clone 6C5) Supplier: Santa Cruz Biotechnology Catalog Number: sc-32233; RRID: AB_627679 Application: Western blot Dilution: 1/10,000<br>anti-GFP Supplier: Abcam Catalog Number: ab290 (RRID:AB_303395) Application: Western blot Dilution: 1/10,000<br>anti-Vinculin (clone VLN01) Supplier: ThermoFisher Scientific Catalog Number: MA5-11690; RRID: AB_10976821 Application: Western blot Dilution: 1/10,000<br>anti-Donkey Anti-Mouse IgG (H+L) Antibody, Alexa Fluor 488 Conjugated Supplier: ThermoFisher Scientific Catalog Number: A-21202 RRID:AB_141607 Application: Fluorescence Western blot Dilution: 1/3000<br>anti-Donkey anti-Rabbit IgG (H+L) Highly Cross-Adsorbed Secondary Antibody, Alexa Fluor 546 Supplier: ThermoFisher Scientific Catalog Number: A10040 RRID:AB_2534016 Application: Fluorescence Western blot Dilution: 1/3000<br>anti-MITF-K206Ac - In house Application: Western blot Dilution: 1/1,000
Validation	anti-HA Validation information: We previously published the result of HA ChIP-seq in non HA-tag expressing 501mel melanoma cell line (https://www.ncbi.nlm.nih.gov/geo/query/acc.cgi?acc=GSE77437). Supplier statement: 'Each lot of Anti-HA antibody is tested for immunoreactivity and purity relative to a reference standard.'

anti-HA (clone HA-7) Supplier Validation information: Test against WB-Cell Line/Tissue Extract. Supplier statement: 'MQ200'
anti-HA (Clone 6E2) Supplier Validation information: 'HA-Tag (6E2) Mouse mAb detects recombinant proteins containing the HA epitope tag. The antibody recognizes the HA-tag fused to either the amino or carboxy terminus of targeted proteins in transfected cells.'
anti-Acetylated-Lysine Supplier Validation information: 'The antibody recognizes acetylated lysine in a wide range of sequence contexts. It has been demonstrated to recognize acetylated histones, p53, CBP, PCAF and chemically acetylated BSA. The antibody has been shown to react with as little as 0.04 ng of chemically acetylated BSA while not recognizing up to 25 µg of nonacetylated BSA.'
anti-MITF (Clone C5) Supplier Validation information: 'Routinely evaluated by Western Blot on Mouse Brain lysates. Control: 501 Mel human melanoma cells, wild-type human, rat, mouse osteoclast cells.'
anti-ERK 2 (Clone K-23) Supplier Validation information: 'affinity purified' and 'Blocking peptide available for competition studies' Catalogue number sc-153 P
anti-ERK 2 (Clone D-2) Supplier Validation information: 'Suitable for use as control antibody for ERK 2 siRNA (h): sc-35335, ERK 2 siRNA (m): sc-35336, ERK 2 shRNA Plasmid (h): sc-35335-SH, ERK 2 shRNA Plasmid (m): sc-35336-SH, ERK 2 shRNA (h) Lentiviral Particles: sc-35335-V and ERK 2 shRNA (m) Lentiviral Particles: sc-35336-V.' and 'Blocking peptide available for competition studies, sc-1647 P'
anti-Thr202/Tyr204, ppERK (Clone 197G2) Supplier Validation information: 'Phospho-p44/42 MAPK (Erk1/2) (Thr202/Tyr204) (197G2) Rabbit mAb detects endogenous levels of p44 and p42 MAP Kinase (Erk1 and Erk2) when dually phosphorylated at Thr202 and Tyr204 of Erk1 (Thr185 and Tyr187 of Erk2), and singly phosphorylated at Tyr204. The antibody does not cross-react with the corresponding phosphorylated residues of either JNK/SAPK or p38 MAP kinase.'
anti-GAPDH (Clone 6C5) SupplierValidation information: 'Suitable for use as control antibody for GAPDH siRNA (h): sc-35448, GAPDH siRNA (m): sc-35449, GAPDH siRNA (r): sc-270067, GAPDH shRNA Plasmid (h): sc-35448-SH, GAPDH shRNA Plasmid (m): sc-35449-SH, GAPDH shRNA Plasmid (r): sc-270067-SH, GAPDH shRNA (h) Lentiviral Particles: sc-35448-V, GAPDH shRNA (m) Lentiviral Particles: sc-35449-V and GAPDH shRNA (r) Lentiviral Particles: sc-270067-V
anti-GFP Supplier Validation information: 'On Western blot the antibody detects the GFP fraction from cell extracts expressing recombinant GFP fusion proteins.' 'Positive control: The Recombinant A. victoria GFP protein (ab84191), any other purified recombinant GFP, any cell line confirmed to overexpress GFP'
anti-Vinculin (clone VLN01) Supplier Validation information: 'Antibody specificity was demonstrated by detection of differential basal expression of the target across cell models owing to their inherent genetic constitution. Relative expression of Vinculin was observed in T-47D, K-562, L6, C2C12, COS-7, MOLT4, Ramos and Jurkat in Western Blot using Vinculin Monoclonal Antibody (Product # MA5-11690).Vinculin is reported to be expressed in T-47D, K-562, L6, C2C12, COS7 and not in other cell lines like MOLT4, Ramos and Jurkat.'
anti-MITF-K206Ac has been validated using peptide array as reported in this study Fig 1e

Eukaryotic cell lines

Policy information about cell lines

Cell line source(s)

The 501mel parental cell line was obtained from Ruth Halaban, Yale (Zakut et al., 1993) and 501mel cells engineered to express ectopic-doxycycline-inducible-HA-tagged-murine-Mitf and HIS-tagged-human-MITF were generated in house for this paper

Authentication

The 501mel cell line has been subject to authentication using STR analysis by Eurofins genomic service

Mycoplasma contamination

All cell lines were routinely check for mycoplasma contamination using the Ludwig Institute routine (monthly) mycoplasma test service using:
Assay kit detail: Lonza MycoAlert® Mycoplasma Detection Kit Catalog # LT07-318 100 tests
Control
MycoAlert® Mycoplasma Detection Kit MycoAlert™ assay control set Catalog # LT07-518

All cell lines were confirmed negative for mycoplasma

Commonly misidentified lines
(See ICLAC register)

None

Animals and other organisms

Policy information about studies involving animals; ARRIVE guidelines recommended for reporting animal research

Laboratory animals

Zebrafish AB/TPL lines were bred and maintained as previously described {Westerfield, 2000 #5199}. Injections were carried out at 1-cell stage embryos and embroys imaged 5 days post-fertilization

Wild animals

No wild animals were used in this study

Field-collected samples

No field-collected samples were used in this study.

Ethics oversight

All zebrafish experiments are performed in accordance with the Animals (Scientific Procedures) Act 1986, and approved by the University of Edinburgh Animal Welfare and Ethical Review Body. Zebrafish AB/TPL lines were bred, raised and maintained as described {Westerfield, 2000 #5199}.

Note that full information on the approval of the study protocol must also be provided in the manuscript.

ChIP-seq

Data deposition

- [x] Confirm that both raw and final processed data have been deposited in a public database such as GEO.
- [x] Confirm that you have deposited or provided access to graph files (e.g. BED files) for the called peaks.

Data access links<br>*May remain private before publication.*	GSE137776<br>https://www.ncbi.nlm.nih.gov/geo/query/acc.cgi?acc=GSE137776
Files in database submission	iMitf_K206Q_0ng_R1_1.fastq.gz<br>iMitf_K206Q_0ng_R1_2.fastq.gz<br>iMitf_K206Q_0ng_R2_1.fastq.gz<br>iMitf_K206Q_0ng_R2_2.fastq.gz<br>iMitf_K206Q_20ng_R1_1.fastq.gz<br>iMitf_K206Q_20ng_R1_2.fastq.gz<br>iMitf_K206Q_20ng_R2_1.fastq.gz<br>iMitf_K206Q_20ng_R2_2.fastq.gz<br>iMitf_K206Q_100ng_R1_1.fastq.gz<br>iMitf_K206Q_100ng_R1_2.fastq.gz<br>iMitf_K206Q_100ng_R2_1.fastq.gz<br>iMitf_K206Q_100ng_R2_2.fastq.gz<br>iMitf_K206Q_Input_1.fastq.gz<br>iMitf_K206Q_Input_2.fastq.gz<br><br>iMitf_K206R_0ng_R1_1.fastq.gz<br>iMitf_K206R_0ng_R1_2.fastq.gz<br>iMitf_K206R_0ng_R2_1.fastq.gz<br>iMitf_K206R_0ng_R2_2.fastq.gz<br>iMitf_K206R_20ng_R1_1.fastq.gz<br>iMitf_K206R_20ng_R1_2.fastq.gz<br>iMitf_K206R_20ng_R2_1.fastq.gz<br>iMitf_K206R_20ng_R2_2.fastq.gz<br>iMitf_K206R_100ng_R1_1.fastq.gz<br>iMitf_K206R_100ng_R1_2.fastq.gz<br>iMitf_K206R_100ng_R2_1.fastq.gz<br>iMitf_K206R_100ng_R2_2.fastq.gz<br>iMitf_K206R_Input_1.fastq.gz<br>iMitf_K206R_Input_2.fastq.gz<br><br>iMitf_WT_0ng_R1_1.fastq.gz<br>iMitf_WT_0ng_R1_2.fastq.gz<br>iMitf_WT_0ng_R2_1.fastq.gz<br>iMitf_WT_0ng_R2_2.fastq.gz<br>iMitf_WT_20ng_R1_1.fastq.gz<br>iMitf_WT_20ng_R1_2.fastq.gz<br>iMitf_WT_20ng_R2_1.fastq.gz<br>iMitf_WT_20ng_R2_2.fastq.gz<br>iMitf_WT_100ng_R1_1.fastq.gz<br>iMitf_WT_100ng_R1_2.fastq.gz<br>iMitf_WT_100ng_R2_1.fastq.gz<br>iMitf_WT_100ng_R2_2.fastq.gz<br>iMitf_WT_Input_1.fastq.gz<br>iMitf_WT_Input_2.fastq.gz<br>GSM4087709_iMitf_K206Q_0ng_R1.ucsc.bedGraph.gz<br>GSM4087710_iMitf_K206Q_0ng_R2.ucsc.bedGraph.gz<br>GSM4087711_iMitf_K206Q_20ng_R1.ucsc.bedGraph.gz<br>GSM4087712_iMitf_K206Q_20ng_R2.ucsc.bedGraph.gz<br>GSM4087713_iMitf_K206Q_100ng_R1.ucsc.bedGraph.gz<br>GSM4087714_iMitf_K206Q_100ng_R2.ucsc.bedGraph.gz<br>GSM4087715_iMitf_K206Q_Input.ucsc.bedGraph.gz<br>GSM4087716_iMitf_K206R_0ng_R1.ucsc.bedGraph.gz<br>GSM4087717_iMitf_K206R_0ng_R2.ucsc.bedGraph.gz<br>GSM4087718_iMitf_K206R_20ng_R1.ucsc.bedGraph.gz<br>GSM4087719_iMitf_K206R_20ng_R2.ucsc.bedGraph.gz

GSM4087720_iMitf_K206R_100ng_R1.ucsc.bedGraph.gz
GSM4087721_iMitf_K206R_100ng_R2.ucsc.bedGraph.gz
GSM4087722_iMitf_K206R_Input.ucsc.bedGraph.gz
GSM4087723_iMitf_WT_0ng_R1.ucsc.bedGraph.gz
GSM4087724_iMitf_WT_0ng_R2.ucsc.bedGraph.gz
GSM4087725_iMitf_WT_20ng_R1.ucsc.bedGraph.gz
GSM4087726_iMitf_WT_20ng_R2.ucsc.bedGraph.gz
GSM4087727_iMitf_WT_100ng_R1.ucsc.bedGraph.gz
GSM4087728_iMitf_WT_100ng_R2.ucsc.bedGraph.gz
GSM4087729_iMitf_WT_Input.ucsc.bedGraph.gz

Genome browser session
(e.g. UCSC)

http://genome-euro.ucsc.edu/s/Pakavarin/iMITF_titration_206_colour_adjusted

Methodology

Replicates

2 biological replicates (each with 30x 15cm dishes) prepared consecutively but all steps post-fixation were carried out in parallel

Sequencing depth

iMit_K206Q_0ng_Dox_Replicate1 Total number of reads=28424720 Uniquely mapped reads=25929609 Av. Input Length=150 paired
iMit_K206Q_0ng_Dox_Replicate2 Total number of reads=30245021 Uniquely mapped reads=27528967 Av. Input Length=150 paired
iMit_K206Q_20ng_Dox_Replicate1 Total number of reads=33702659 Uniquely mapped reads=30779108 Av. Input Length=150 paired
iMit_K206Q_20ng_Dox_Replicate2 Total number of reads=32900533 Uniquely mapped reads=30009364 Av. Input Length=150 paired
iMit_K206Q_100ng_Dox_Replicate1 Total number of reads=27100903 Uniquely mapped reads=24741618 Av. Input Length=150 paired
iMit_K206Q_100ng_Dox_Replicate2 Total number of reads=31639213 Uniquely mapped reads=28845804 Av. Input Length=150 paired
iMit_K206Q_Input_control Total number of reads=38542817 Uniquely mapped reads=35227922 Av. Input Length=150 paired

iMit_K206R_0ng_Dox_Replicate1 Total number of reads=31555438 Uniquely mapped reads=28798671 Av. Input Length=150 paired
iMit_K206R_0ng_Dox_Replicate2 Total number of reads=31352594 Uniquely mapped reads=28684192 Av. Input Length=150 paired
iMit_K206R_20ng_Dox_Replicate1 Total number of reads=33733351 Uniquely mapped reads=30941219 Av. Input Length=150 paired
iMit_K206R_20ng_Dox_Replicate2 Total number of reads=31079871 Uniquely mapped reads=28520630 Av. Input Length=150 paired
iMit_K206R_100ng_Dox_Replicate1 Total number of reads=30973546 Uniquely mapped reads=28344856 Av. Input Length=150 paired
iMit_K206R_100ng_Dox_Replicate2 Total number of reads=33097713 Uniquely mapped reads=30396518 Av. Input Length=150 paired
iMit_K206R_Input_control Total number of reads=31162561 Uniquely mapped reads=28473720 Av. Input Length=150 paired

iMit_WT_0ng_Dox_Replicate1 Total number of reads=23821343 Uniquely mapped reads=21786519 Av. Input Length=150 paired
iMit_WT_0ng_Dox_Replicate2 Total number of reads=27099478 Uniquely mapped reads=24805484 Av. Input Length=150 paired
iMit_WT_20ng_Dox_Replicate1 Total number of reads=29491670 Uniquely mapped reads=26928341 Av. Input Length=150 paired
iMit_WT_20ng_Dox_Replicate2 Total number of reads=25850947 Uniquely mapped reads=23689580 Av. Input Length=150 paired
iMit_WT_100ng_Dox_Replicate1 Total number of reads=34846438 Uniquely mapped reads=32066253 Av. Input Length=150 paired
iMit_WT_100ng_Dox_Replicate2 Total number of reads=36319862 Uniquely mapped reads=33368844 Av. Input Length=150 paired
iMit_WT_Input_control Total number of reads=31351213 Uniquely mapped reads=28567297 Av. Input Length=150 paired

Antibodies

anti-HA (clone 12CA5) Supplier: Roche Catalog Number: 11666606001; RRID: AB_514506

Peak calling parameters

Mapping
STAR --runThreadN 24 --genomeDir /t1-data/user/plouphra/STAR_Genome_hg38/ --readFilesIn "$1" "$2" --outFileNamePrefix "$3" --readFilesCommand zcat -c --chimSegmentMin 20 --outReadsUnmapped Fastx
"$1" = *_1.fastq.gz
"$2" = *_2.fastq.gz
"$3" = output file name prefix

Peak calling & annotation
makeTagDirectory "$1" -genome /t1-data/user/plouphra/hg38/ "$2"
findPeaks "$1" -style factor -norm 3e7 -i "$3" -o "$1"_peaks.txt
annotatePeaks.pl "$1"/peaks.txt -genome /t1-data/user/plouphra/hg38/ -CpG -gsize 2790000000 > "$1"/annotated_peaks.txt -go "$1"/GO.txt -genomeOntology "$1"/genomeOntology.txt

"$1" = samples name/directory e.g. iMitf_WT_0ng_R1
"$2" = input SAM file output from STAR
"$3" = tag_directory generated from STAR-mapped SAM files of the input control samples

Generating normalised Bedgraph for UCSC
makeUCSCfile "$1" -fsize 1e50 -o auto

Data quality

All peaks were called using the following fixed parameters
FDR rate threshold = 0.001000000
Fold over input = 4.00
Poisson p-value over input = 1.00e-04
Fold over local region = 4.00
Poisson p-value over local region = 1.00e-04
and variable parameters (modelled according to each dataset as these are sequencing depth dependent)
FDR effective poisson threshold
FDR tag threshold

iMit_K206Q_0ng_Dox_Replicate1 # FDR effective poisson threshold = 3.145650e-06 # FDR tag threshold = 17.0 Fragment length=196 Peaks called=78579 Differential peaks over input (filtered)=74467 Local background filtering (filtered)= 2145 Clonal filtering (filtered)=0 ChIP-efficiency=0.24% total peaks (passed) = 1967

iMit_K206Q_0ng_Dox_Replicate2 # FDR effective poisson threshold = 2.514935e-06 # FDR tag threshold = 18.0 Fragment length=210 Peaks called=82617 Differential peaks over input (filtered)=78729 Local background filtering (filtered)= 2288 Clonal filtering (filtered)=0 ChIP-efficiency=0.17% total peaks (passed) = 1600

iMit_K206Q_20ng_Dox_Replicate1 # FDR effective poisson threshold = 3.296687e-06 # FDR tag threshold = 19.0 Fragment length=210 Peaks called=82641 Differential peaks over input (filtered)=75944 Local background filtering (filtered)= 2011 Clonal filtering (filtered)=0 ChIP-efficiency=1.00% total peaks (passed) = 4686

iMit_K206Q_20ng_Dox_Replicate2 # FDR effective poisson threshold = 2.638019e-06 # FDR tag threshold = 19.0 Fragment length= 204 Peaks called= 85601 Differential peaks over input (filtered)= 78454 Local background filtering (filtered)= 2083 Clonal filtering (filtered)= 0 ChIP-efficiency=1.12% total peaks (passed) = 5064

iMit_K206Q_100ng_Dox_Replicate1 # FDR effective poisson threshold = 2.834221e-06 # FDR tag threshold = 17.0 Fragment length= 198 Peaks called=77637 Differential peaks over input (filtered)= 71864 Local background filtering (filtered)= 2199 Clonal filtering (filtered)= 0 ChIP-efficiency= 0.67% total peaks (passed) = 3574

iMit_K206Q1010ng_Dox_Replicate2 # FDR effective poisson threshold = 2.492754e-06 # FDR tag threshold = 18.0 Fragment length= 206 Peaks called= 88142 Differential peaks over input (filtered)= 81656 Local background filtering (filtered)=2589 Clonal filtering (filtered)= 0 ChIP-efficiency=0.72% total peaks (passed) = 3897

iMit_K206R_0ng_Dox_Replicate1 # FDR effective poisson threshold = 7.334091e-06 # FDR tag threshold =17.0 Fragment length= 204 Peaks called= 106808 Differential peaks over input (filtered)= 88369 Local background filtering (filtered)= 10058 Clonal filtering (filtered)= 0 ChIP-efficiency=0.99% total peaks (passed) = 8381

iMit_K206R_0ng_Dox_Replicate2 # FDR effective poisson threshold =5.694232e-06 # FDR tag threshold =17.0 Fragment length= 204 Peaks called= 105830 Differential peaks over input (filtered)=86841 Local background filtering (filtered)= 10386 Clonal filtering (filtered)= 0 ChIP-efficiency=0.99% total peaks (passed) = 8603

iMit_K206R_20ng_Dox_Replicate1 # FDR effective poisson threshold = 1.332323e-05 # FDR tag threshold = 19.0 Fragment length=206 Peaks called=148494 Differential peaks over input (filtered)=84809 Local background filtering (filtered)=9536 Clonal filtering (filtered)= 0 ChIP-efficiency=11.98% total peaks (passed) = 54149

iMit_K206R_20ng_Dox_Replicate2 # FDR effective poisson threshold = 5.147797e-06 # FDR tag threshold =19.0 Fragment length=198 Peaks called=128399 Differential peaks over input (filtered)= 68606 Local background filtering (filtered)= 7919

Clonal filtering (filtered)= 0 ChIP-efficiency=11.87% total peaks (passed) = 51874

iMit_K206R_100ng_Dox_Replicate1 # FDR effective poisson threshold =5.961963e-06 # FDR tag threshold = 19.0 Fragment length=218 Peaks called= 124891 Differential peaks over input (filtered)= 59170 Local background filtering (filtered)=7304 Clonal filtering (filtered)= 0 ChIP-efficiency=14.23% total peaks (passed) = 58417

iMit_WT_0ng_Dox_Replicate1 # FDR effective poisson threshold = 3.651436e-06 # FDR tag threshold =17.0 Fragment length= 208 Peaks called= 73101 Differential peaks over input (filtered)= 55932 Local background filtering (filtered)= 4323 Clonal filtering (filtered)= 0 ChIP-efficiency=2.55% total peaks (passed) = 12845

iMit_WT_0ng_Dox_Replicate2 # FDR effective poisson threshold = 5.008143e-06 # FDR tag threshold = 18.0 Fragment length= 202 Peaks called= 81440 Differential peaks over input (filtered)= 65900 Local background filtering (filtered)=4598 Clonal filtering (filtered)= 0 ChIP-efficiency=2.03% total peaks (passed) = 10942

iMit_WT_20ng_Dox_Replicate1 # FDR effective poisson threshold = 9.104248e-06 # FDR tag threshold = 19.0 Fragment length= 210 Peaks called= 116466 Differential peaks over input (filtered)=65761 Local background filtering (filtered)= 6358 Clonal filtering (filtered)= 0 ChIP-efficiency=9.54% total peaks (passed) = 44347

iMit_WT_20ng_Dox_Replicate2 # FDR effective poisson threshold = 7.311743e-06 # FDR tag threshold = 18.0 Fragment length= 220 Peaks called= 113694 Differential peaks over input (filtered)= 55696 Local background filtering (filtered)= 6124 Clonal filtering (filtered)= 0 ChIP-efficiency=13.17% total peaks (passed) = 51874

iMit_WT_100ng_Dox_Replicate1 # FDR effective poisson threshold = 6.461421e-06 # FDR tag threshold = 21.0 Fragment length= 224 Peaks called= 142484 Differential peaks over input (filtered)=53281 Local background filtering (filtered)=7108 Clonal filtering (filtered)= 0 ChIP-efficiency=22.87% total peaks (passed) = 82095

iMit_WT_100ng_Dox_Replicate2 # FDR effective poisson threshold = 1.289524e-05 # FDR tag threshold = 21.0 Fragment length= 224 Peaks called= 148377 Differential peaks over input (filtered)= 61584 Local background filtering (filtered)= 7356 Clonal filtering (filtered)= 0 ChIP-efficiency=21.78% total peaks (passed) = 79437

Software

STAR v 2.5.1b, Genome GRCh38 v23 (Dobin et al., 2013)

Homer v4.9.1, Genome hg38 v5.10 (Heinz et al., 2010)

ggplot2-v3.0.0 – under R-v3.5.1 https://CRAN.R-project.org/package=ggplot2

TreeView v1.1.6r4 (Saldanha, 2004)

PicardTools version 1.96,   http://picard.sourceforge.net

MEME v5.0.1 (Machanick and Bailey, 2011)

For the fluorescence anisotropy experiments:
Data collection was performed using Tecan i-control (Version 1.10.4.0)

Data fitting was performed using GraphPad Prism (Version 9.3.1)
